# Genome Sequence of *Acidovorax avenae* Strain T10_61 Associated with Sugarcane Red Stripe in Argentina

**DOI:** 10.1128/genomeA.01669-15

**Published:** 2016-02-04

**Authors:** Paola D. Fontana, Cecilia A. Fontana, Daniela Bassi, Edoardo Puglisi, Sergio M. Salazar, Graciela M. Vignolo, Pier S. Coccocelli

**Affiliations:** a INTA EEA Famaillá, Tucumán, Argentina; b Istituto di Microbiologia, Università Cattolica del Saco Cuore, Cremona-Picenza, Italy; c CERELA, CONICET, Tucumán, Argentina

## Abstract

Red stripe of sugarcane in Argentina is a bacterial disease caused by *Acidovorax avenae*. The genome sequence from the first isolate of this bacterium in Argentina is presented here. The draft genome of the *A. avenae* T10_61 strain contains 5,646,552 bp and has a G+C content of 68.6 mol%.

## GENOME ANNOUNCEMENT

The bacterium *Acidovorax avenae* causes disease in a wide range of economically important monocotyledonous and dicotyledonous plants, including sugarcane, corn, rice, oats, foxtail millet, watermelon, and orchids (1).

In Argentina the red stripe disease of sugarcane caused by this bacteria affects 30% of the milling stems and consequently the juice quality, with important economic losses. In the last decade, due to new agricultural techniques such as green-cane harvesting and crop rotation with soybean, an increase in the incidence of this infection was observed. Despite the documentation of significant production losses, red stripe disease has been poorly studied. Fontana et al. (2) reported for the first time the isolation, identification, and molecular characterization of *A. avenae* in Argentina. 

In this work *de novo* shotgun sequencing of the *A. avenae* T10_60 strain, isolated from sugarcane leaves with typical symptoms of red stripe in Tucumán-province of Argentina, was performed. The whole genome was sequenced using an Illumina MiSeq sequencing system. Quality-filtered reads were assembled using Velvet software (version 1.1.04) (3), which generated 130 contigs. The draft genome sequence of strain T10_61 comprises 5,646,552 bases, representing approximately 99.9% of the estimated genome of this strain. The RAST server annotation (4) revealed that this genome includes approximately 5,096 coding sequences (CDSs) and 58 tRNAs and contains 480 subsystems. The genome of this strain has a high G+C content, 68.6%. Putative functions of encoding genes were automatically identified using RAST. Further analyses of genome sequence data are in progress to better understand the pathogenic potential of this microorganism as well as comparative studies with the genomes of others *A. avenae* strains from different sources.

### Nucleotide sequence accession number.

This whole-genome shotgun project has been deposited at DDBJ/EMBL/GenBank under accession number LJGO00000000. The version described in this paper is the first version.
