# Erratum for Fontana et al., Genome Sequence of *Acidovorax avenae* Strain T10_61 Associated with Sugarcane Red Stripe in Argentina

**DOI:** 10.1128/genomeA.00202-16

**Published:** 2016-03-17

**Authors:** Paola D. Fontana, Cecilia A. Fontana, Daniela Bassi, Edoardo Puglisi, Sergio M. Salazar, Graciela M. Vignolo, Pier S. Cocconcelli

**Affiliations:** a INTA EEA Famaillá, Tucumán, Argentina; b Istituto di Microbiologia, Università Cattolica del Sacro Cuore, Cremona-Piacenza, Italy; c CERELA, CONICET, Tucumán, Argentina

## ERRATUM

Volume 4, no. 1, e01669-15, 2016. Page 1: The byline should read as given above.

